# The Sanitary Regulations of Antient Rome
*Juvenalis (D. J.) Mores Hominum. Ed. Sir R. Stapylton. London: 1660.Eutropii, Historia Romana. C. Bradley. London: 1826.Vitruvius, De Architecturâ. Venet: 1511.Frontinus, De Aquæductibus. Romæ, circa 1486.


**Published:** 1855-12

**Authors:** Alfred Haviland


### REVIEWS.

### (CRITICAL, ANALYTICAL, AND HISTORICAL.)

### THE SANITARY REGULATIONS OF ANTIENT ROME.*

PART III.

### III.—The Regulations for the Cleanliness and Ventilation of Houses and Streets, Drainage, Windows, Sleeping Apartments, Doors, Height and Aspect of Dwellings, Number of Houses and Inhabitants.

Perhaps in the history of the mightiest city or the humblest village, there are few things more remarkable than the gradual changes which take place in the buildings composing them. Gradual, however, as these are as a general rule, yet we must concede that the most perfect have been those that extensive conflagrations have brought about. Many of the grandest cities have at one time or another been destroyed, either by accident or design. Rome suffered at the hands of the besieging Gauls, and then was again destroyed by one of its own emperors. After each apparent disaster improvements took place, which soon wiped from the memory of the citizens any regret that they might have felt, when beholding their well known homes consumed by the flames. For hundreds of years the conquerors of the world were contented with small and close houses, roofed either with thatch or shingles. After Lepidus, B.C. 78, had introduced Numidian marble, and at the time when luxury was beginning to take the place of simple habits, a desire to have splendid edifices soon rose to a great height. How great the difference between the thatched hut and the house of Autronius, that cost the consul Messalla 3700 sestertia, about 33,000*l.* At first the houses were only one story high; but as in our modern cities, the land on which these houses were built became expensive, so did the proprietors increase the number of the stories, until the formidable height of some of the houses was the cause of serious accidents, which induced the emperor Augustus to restrict the height to seventy feet, of all houses built near a thoroughfare.

The bed-rooms were generally small; and as the means of heating houses were very inefficient among the Romans, these apartments were generally built in that part of the house where the sun had the greatest influence. Vitruvius recommended that the dormitories should look towards the east, in order to have the benefit of the first rays of day. Generally, however, the bed-rooms had merely the borrowed light derived from the openings in the upper part of

---

* Juvenalis (D. J.) Mores Hominum. Ed. Sir R. Stapylton. London: 1660.
Eutropii, Historia Romana. C. Bradley. London: 1826.
Vitruvius, De Architecturâ. Venet: 1511.
Frontinus, De Aquæductibus. Romæ, circa 1486.

the house, which lighted the atrium—a large apartment, roofed over, with the exception of an opening in the centre (compluvium), through which the rain fell from the sloping roof into a cistern on the floor (impluvium). This peculiar arrangement must have been highly conducive to the free ventilation of the house; besides which, the defective windows, which were for many centuries merely closed by shutters, not only admitted of the free ingress and egress of air, but also acted as chimneys for the smoke from portable furnaces or braziers, in which charcoal or coal were burnt. The rooms were sometimes heated by hot water, conveyed from furnaces into pipes. It must be remembered that, at a later date, mica, lapis specularis, and even glass, were used for windows; and hence their name, specularia. Chimneys seem to have been entirely unknown, or at least very imperfect. The Romans were eminently fond of being out of doors; and we find that many houses were provided with solaria on their tops, which were intended for basking in the sun. Many facts connected with the social life of the Romans, lead us to account for what is remarkable in the construction of their sleeping apartments. Their fondness for exercise in the open air especially attracts attention, for all well know how conducive it is to sound repose, during which the smallness of the bed-room would be forgotten. The atrium was frequently used as a family sitting-room. So far, then, as the ventilation of Roman houses in general is concerned, we may safely assert that their construction was highly conducive to a thorough and continued renewal of the air of the apartments. The compluvium, the open ceilings, the windows, and the means of heating their principal rooms, all conspired to bring about a process which, in our more complicated dwellings, seldom is effective. Of their *drainage*, we shall presently speak.

The floors of the houses were generally formed of some solid brick or tile work. Sometimes they were composed of fragments of marble or brick, admixed with mortar and beaten down. Marble, in the luxurious period, was a very favourite material used for flooring. Wood was not made much use of.

The above description of houses applies to the *domus*, and before entering upon the population, it may be as well to remark, that the lofty houses above mentioned, contained in their *cœnacula* or apartments, many families, the lodging-house itself was called *insula*. The close crowding of these places are often noticed by the writers of the day. Juvenal, in his third Satire, gives his reasons for preferring a house in the country to a town lodging, where he says diseases are bred, and sleep is denied to the poor artizan, whose room is near the street, on account of the noises of the carmen. With all the inconveniences with which these *insulæ* abounded, they produced an annual rent of nearly three or four hundred pounds (40,000 sesterces).

Codrus the poet, with his wife, had a wretched apartment three stories high, just underneath the tiles, where, as Juvenal remarks, they had not only to fear the fire from below, but the rain from above. The unequal division of the surface of the ground of the city between the rich and the poor, was thus the cause of the very evil which has ever subsisted in large cities. Whilst the one luxuriated in the well ventilated and capacious *domus*, the other was obliged to put up with the loathsome *cœnacula* of the *insulæ*, subject to all the miseries of close confinement, to which were superadded the constant dread of being all burnt out (for fires were so constant at Rome, that the Gabian or Alban stone was ordered to be used for certain parts, as it was considered fire-proof), or else drowned out by the rain pouring through an imperfect roof.

*Population and Number of Houses.* Eutropius says that under Servius Tullius, B. C 540, who was the first to command a *census* to be made, there were near 84,000 Roman citizens, including those who worked in the fields. The census was established at Rome in order to estimate *(censere)* the amount of property enjoyed by the citizens: it was generally held about once in five years. In the time of Theodosius there were 48,382 houses, of which there were 1710 *domus* or respectable houses and mansions, and 46,602 *insulæ*, inhabited by the lower order. According to the estimate made in Paris, 25 persons were allotted to each house; and if this be applied to Rome, the amount of population at this period must have been about between eleven and twelve hundred thousand. With rcgard to the populousness at earlier periods, history has not furnished data on which to depend; the above statement, however, in all probability, approximates the truth, and affords a strong contrast to the last returns, which show that Rome contains within its walls only 224,000, or hardly one-fifth of the number.

*The Drainage of Rome.* The sewers of Rome were called *cloacæ;* the main drain *(cloaca maxima)* was ascribed to Tarquinius Priscus. Strabo said that a cart laden with hay could easily traverse it; and Dion Cassius states that Agrippa, when he cleansed the sewers, passed through them in a boat. The main drain opened into the Tiber; it received all the lesser drains from the houses, and apertures were made in the streets at proper distances for the reception of dirty water, etc. These sewers were supported by public funds, and under the superintendence of a regular police, called *curatores cloacarum.*

*The Streets of Rome.* These were of all descriptions, from the narrow lanes and *culs-de-sac* (*angiportus*), to the main streets, of which each division of the city had at least one. The streets were generally well paved, either with blocks of basaltic lava, or else with gravel and travertine. They varied at different epochs; at one time, when the population was comparatively scanty, they were bordered by houses only one story high; in later times, when Rome became an overgrown city, they were overshadowed by high habitations, which had the effect of impeding evaporation and obstructing the sun's rays. The streets were raised slightly in the centre, so as to admit of the rain flowing on each side into the drains. Like those of our own metropolis, the streets of Rome were greatly improved after the city had been partially burnt down. Before the conflagration by the Gauls, the streets were narrow and irregular; and after that event, the city was so hastily rebuilt, that it was in this respect but slightly improved. After* its destruction by fire, however, during the reign of Nero, it was rebuilt with great regularity, the streets were widened, and in a variety of respects due regard was paid to the comfort and health of the inhabitants, so that really some considerable advantage was derived from this freak, if freak it can be called, of the Emperor; although some have supposed that the wider streets and the lower houses did not improve the salubriousness of the city, in consequence of this arrangement subjecting the people to the full rays of a hot sun.

### IV. The Means used to keep the Physical Powers of the Romans as perfect as possible. Exercises, Games, Marriage, Employment of Time, Construction of large Buildings, and Exercises in the Army.

*Exercises and games.* The Romans being an eminently warlike people, their physical education was particularly cared for, and everything that conduced to health, strength, and activity, was strictly enjoined to be adopted by the rulers of the people, whose habits and tastes led them, as it were instinctively, to obey those wise regulations. The army was the great school of the Roman; there every species of exercise was employed; in fact, the term itself, *exercitus*, had its origin in the Latin verb which means to *exercise;* but in the private life of the civilian also a due regard to the care of the body was maintained. The fore part of the day was entirely given up to business; after which, pleasure and relaxation were sought. Among some of the favourite games, were the play at tennis, or with the hand-ball, whilst others walked or danced; jumping, running, vaulting, were also practised. In more luxurious times, covered gardens were constructed, in order to insure exercise at all seasons: these, according to Vitruvius, were open towards the south in the winter, and the north in the summer. The Campus Martius was the favourite resort of the antient Romans; there they rode on horseback, practised the use of the dart and the bow, played at quoits and a thousand other games, after which they retired to the baths, attached to which were often *gymnasia* and *palestræ*, where regular trainers attended and taught boxing, wrestling, and every other manly exercise. These healthful exercises, from being merely intended to ensure health, at last, however, were carried to a great excess, of which the gladiatorial, and other equally disgraceful exhibitions, were the result. Leaping was especially taught among the soldiery, as it enabled the foot soldier to encounter difficulties in the line of march, pursuit or flight, which otherwise would baffle him. Many of the manœuvres during

* Gell. Top. of Rome, pp. 40, 41.

drill, were made by the soldier equipped in the heaviest possible manner, in order that, when in actual service, he might feel the benefit of being only lightly laden. Those men who conducted the exercises of the army were called *campidoctores.* The Romans ever entertained the very sensible opinion that exercise was what Asclepiades termed it, *the common aid of physic;* and acting upon this belief, the gymnasia were dedicated to Apollo, the god of physicians. Celsus, we find, made himself exceedingly popular by his great invention of exercises, whereby he made his rules for attaining health both agreeable and useful: the hanging beds were his invention. When the Roman physicians ordered their consumptive patients off to Alexandria, the exercise consequent upon such a journey was considered of more remedial value than the change of air. Galen laid great stress upon the use of the *strigil.* In the gymnasium were to be found various officers; some looked after wounds, dislocations, etc., whilst others gave advice on regimen, or administered clysters and medicines. Abstemiousness was ever considered highly necessary for the athlete and for the man who employed exercise as a remedial agent.

With regard to the sanitary regulations in the army, little more can be added, for much the same system was adopted by the soldiery as we have found to be in vogue among the civilians; in fact, the latter derived many of their exercises and games from the former. The soldier's health was at all times a matter of great concern to the state, and many wise provisions were doubtlessly made, although our information on this point is scanty. When in active warfare, the legions during the winter were withdrawn into some friendly city, or distributed among villages, in order to protect them from the inclemency of the season; and to order an army, except in case of necessity, to pass the winter in their tents, was done as a severe punishment. There were *summer* encampments and *winter* encampments; huts were constructed in the *hiberna* of turf or stone. The selection of the ground for the camp was considered of the highest importance; facilities for obtaining, without being subject to assault, wood, water and forage, was ever deemed of vital consequence; in fact, the position of the tent was always made in regard to this first consideration. The soldiers and centurions rose at daybreak and received their orders. The *valetudinarium* was an apartment set aside as a hospital for sick and wounded soldiers; the *veterinarium* received the sick horses. The Roman camp, like everything else, degenerated; the soldiers were crowded together in half the space that they were allowed under the republic, and in consequence of a prevailing apathy in later times, the defences were but indifferently thrown up.

*Marriage.* Whatever tends to prevent or check vice is a sanitary regulation, inasmuch as immorality has ever for its sequel some physical or mental ill, which has either an immediate or a remote effect upon the community. Nature being the great guide for rulers,—whether they be fathers of families or heads of states,

—will be ever ready with her direst revenge, if they allow her pure voice to remain unheeded, and permit art to trammel her with senseless restrictions, opposed as they are both to religion and morality. The number of marriages is generally the criterion of a country's prosperity; for the fluctuations in their statistics will generally indicate some disturbances in the even tenour of a nation's welfare and happiness. Peace and prosperity encourage it; whilst war, pestilence, and famine, have an opposite effect. The Romans, when comparatively natural and healthy, were great advocates of marriage—witness the rape of the Sabines; they enjoined it by their example, and afterwards enforced it by penalties. The Roman at a later period, when art had triumphed over nature, preferred the sickly licentiousness of adultery and concubinage to the pure domestic *placens uxor* of his forefathers. Still, amidst all the dreadful carelessness that reigned after her wealth had become incalculable, the simple instruction which Rome received in her youth was not altogether forgotten; for she still endeavoured to remedy the evil by imposing heavy penalties upon unmarried men, reminding us forcibly of some profligate in his wild career of vice, recalling amidst his revels the pure prayer which his mother had taught him when young and innocent. As with individuals, so is it with nations. By law puberty was considered to be attained at the fourteenth year in the male, and at the twelfth year in the female.

It is evident that the Romans ever had in view the increase of the population; for we find that the penalties on cœlibes ceased when they had attained a certain age, which, so far as the woman was concerned, was considered incompatible with fecundity. Registration of children took place under M. Antoninus, who ordered that children should be registered by name within thirty days from their birth. Enough is said, so far as matrimony is concerned as a sanitary regulation, when we assert from history that the Romans did all they could to encourage it, by honouring those who were wise enough to fulfil the dictates of nature and the state, and by heaping pains and penalties upon those who foolishly forfeited natural happiness for artificial pleasure.

*Employment of Time, and Construction of large Buildings.* A nation that has its fortune to make is generally healthy, active, and resilient; such was the case with Rome. In its early history we find the Romans ever on the alert, some great work to be achieved, some national undertaking absorbing the public mind. In fact, the constant employment of the minds and bodies of the Romans kept their state prosperous; the aqueducts, whose construction required thousands of hands, not only gave food to myriads whilst they were being formed, but afforded health to all the citizens when complete. Then let us review the public buildings, how much concentration of talent is there evidenced, even in the ruins! The Irishman's curse—"May the spirit of building come upon you"—was inapplicable as a malediction in the good old times of antient Rome.

The decline and fall of the Roman empire was dependent upon the change that took place in the health of the minds and bodies of the people. The Romans became degenerate in their affluence and idleness, for they had nothing to look forward to, all had been done for them; their fortunes were made, and their fortune proved their ruin. The mind of an Englishman is ever on the alert; he has science to interest him, and this alone will ever find new objects for his study. The Roman knew not science as we know it; so soon therefore as his city was complete with everything that could gratify his sensual taste, that city fell.

### V. THE MEANS USED TO WARD OFF EPIDEMICS.

*Religion*: *Physicians: Esculapius.* For centuries Rome was without physicians; and the plagues that visited her in the form of epidemics were ever considered, as they ever will be, as the effect of the wrath of heaven; consequently we find that all manner of superstitious rites were instituted and performed for the sake of appeasing the wrath of their gods. B.C. 462, a day of public humiliation was appointed, and every species of public and private supplication was offered up to stay the devastating disease. B.C. 433, solemn prayer and the Sybilline books were had recourse to. Votive offerings were made in the temple of Apollo, B.C. 430, and at the same time the Sybilline book was consulted. B.C. 396, in conjunction with the usual form of taking out the Sybilline books a new rite was instituted, called the *Lectisternium*, which was kept up for days. In B.C. 362, after everything had failed, a dictator was appointed to drive a nail into the door of the temple of Jupiter. The physicians seem to have suggested nothing; in fact, we find that in the year B.C. 293, which, from being one of rejoicing, was turned into one of sorrow by pestilence, the volumes of the Sybil were consulted as usual, and the statue of Esculapius was filched from Epidaurus by the Romans and placed in their city.

### VI. MODE OF BURIAL.

*Funerals and Burials.* With us these terms are accepted as almost synonymous; not so, however, were they in antient Rome: at one time the Romans buried their dead, at another they wisely burnt them; at all times, however, I believe the Pagans considered that, where the dead were buried, the place was sacred (*religiosus*), and thus afforded a good example to our present Christian Church restorers, against whom, I fear, the *sepulcri violati actio* could be too often brought.

The Romans, like many other nations, followed rather their instincts than their reason in many of their great provisions for self-preservation. The badger instinctively avoids befouling his own burrow; and what may be said of the badger may be affirmed of many birds and animals—man excepted. In his *instinctive* state he, however, has been found to avoid the stigma which the well known proverb contains. To return, however, to the Romans: they buried their dead at first, but *without* the city; they then, during the empire, when the population was great, almost without exception *burnt* their dead, and the ashes were disposed of in urns, which were placed outside the city; only a favoured few were allowed to find their last resting place within the walls. Illustrious men were, however, buried in the Campus Martius and Campus Esquilinus; but these were not in the heart of the city. Even, however, this limited intramural burial was considered unhealthy; and the place was awarded to Mæcenas, who laid it out in gardens, and built thereon a splendid residence. Yet Horace leads us to infer that other than the illustrious were buried therein:

"In coffins still the herd of slaves
Were hither brought to crowd the graves;
And once in this detested ground
A common tomb the vulgar found;
Buffoons and spendthrifts, vile and base,
Together rotted here in peace."*

As a general rule the bodies or their ashes were deposited by the roadside, so that some of the roads were lined with the tombs of all classes, for miles in extent. Is it not at once evident, that this was a most wise sanitary regulation? Among the Romans a dead body was looked upon as an eminent source of pollution.

We have thus endeavoured to present to our readers a sketch of those institutions which, in the early history of Rome, either directly or indirectly affected the health of her citizens. In many respects Rome was unhappily situated, for the water was bad, the soil volcanic, the river Tiber contaminated by mineral waters, and the plain from which the Seven Hills arose generated malarious gases; yet with all these natural disadvantages she contended successfully, and her ruins now remain to tell the tale of her former grandeur, and the indomitable energy of her people, who learnt to conquer the world by being nurtured amidst difficulties themselves.

*Alfred Haviland.*

---

### FUEL IN ITS SANITARY APPLICATIONS.†

* Satires. Book I, Sat. viii, l. 16. Francis.

